# Brand-specific enhanced safety surveillance of GSK’s Fluarix Tetra seasonal influenza vaccine in England: 2017/2018 season

**DOI:** 10.1080/21645515.2019.1705112

**Published:** 2020-03-02

**Authors:** Simon de Lusignan, Silvia Damaso, Filipa Ferreira, Rachel Byford, Christopher McGee, Sameera Pathirannehelage, Vishvesh Shende, Ivelina Yonova, Alexander Schmidt, Anne Schuind, Gael Dos Santos

**Affiliations:** a University of Surrey, Guildford, UK; b Royal College of General Practitioners (RCGP) Research and Surveillance Centre (RSC), London, UK; c GSK, Wavre, Belgium; d VPN Consultancy Limited (on behalf of GSK), London, UK; e GSK, Rockville, MD, USA

**Keywords:** Safety management, medical records systems, adverse reactions, influenza vaccines, general practice, England

## Abstract

In compliance with the European Medicine Agency guidance to detect any potential safety concerns associated with influenza vaccination, an enhanced safety surveillance study was conducted in England during the 2017/18 influenza season. The primary objective was to estimate the incidence rates of adverse events occurring within seven days of vaccination with Fluarix Tetra. In nine General Practices, seasonal influenza vaccine was administered to patients according to local guidelines. Events following immunization were collected using customized cards (enhanced component) combined with electronic health records [EHRs] (EHR component) to estimate incidence rates of adverse events experienced post vaccination. The study ran from 01-Sep-2017 to 30-Nov-2017. A total of 23,939 subjects were vaccinated of whom 16,433 received Fluarix Tetra. The cumulative incidence rates of adverse events of interest for Fluarix Tetra were 7.25% [95% CI, 5.95–8.73] for events reported by card alone, and 9.21% [95% CI, 7.37–11.34] when combined with EHR data. The type and frequency of events reported were consistent with the Fluarix Tetra Summary of Product Characteristics. The study supports and confirms the safety profile of Fluarix Tetra.

ClinicalTrials.gov number: NCT03278067

**Plain language summary**

***What is the context?***

Influenza disease is an acute viral respiratory infection. Seasonal influenza has a substantial health and economic impact every year. Vaccination remains the primary prevention strategy. Since the influenza virus evolves continuously, the vaccine needs to be frequently adjusted. Influenza vaccination programs are implemented over a short period of time and many individuals receive the vaccine in a short period of time, increasing the need for regular and timely assessment. The European Medicines Agency requires influenza vaccine manufacturers to implement annual surveillance called ‘enhanced safety surveillance’. The aim of this requirement is to rapidly detect any increase in the frequency or severity of adverse reactions following seasonal influenza vaccination.

***What is new?***

An ‘enhanced safety surveillance’ study has been conducted in England in 2017–2018. In this study, electronic health records of registered patients were used to evaluate the safety of the vaccine in different age groups and in subjects with different risk level for the disease. To collect adverse reaction experiences more exhaustively, reporting cards were used.

***What is the impact?***

In this study, no adverse reactions were detected which could impact the benefit of influenza vaccination from a public health perspective. The vaccine safety profile was similar regardless of the health status of the people vaccinated. The enrolled population essentially covered groups targeted for vaccination, showing that the vaccine’s recommendations in England were followed appropriately.

## Introduction

Influenza has a significant clinical and economic impact worldwide, with an estimated 290,000–650,000 seasonal influenza-associated respiratory deaths per year globally; the number would be substantially higher if deaths from other influenza-related diseases such as cardiovascular disease were taken into account.^1^ According to the European Center for Disease Control, seasonal influenza causes 4–50 million symptomatic cases in the European Union/European Economic Area (EU/EEA) annually, with 15,000–70,000 deaths from causes associated with influenza.^2^ Immunization remains the primary public health prevention mechanism, but because the influenza virus undergoes genetic and antigenic changes, the vaccines are regularly reformulated following the recommendations of the World Health Organization (WHO).^3^

Seasonal influenza vaccines are used over a relatively short time period, with vaccination campaigns starting prior to peak influenza activity, which generally occurs in January or February in the Northern Hemisphere. In the United Kingdom (UK), groups eligible for influenza vaccination are based on the advice of the Joint Committee on Vaccination and Immunization (JCVI), with the intent to provide direct protection to individuals at higher risk of influenza-associated morbidity and mortality. This encompasses older people (aged 65 and over), those with existing underlying medical conditions (since 2010), pregnant women and (since 2012) healthy children.^4-7^ The regular evolution of influenza viruses and reformulation of vaccines warrant constant benefit-risk monitoring. To that end, the European Medicines Agency (EMA) Pharmacovigilance Risk Assessment Committee (PRAC) released a guidance document listing the requirements for annual enhanced safety surveillance (ESS). The objective is to rapidly detect any increased local and systemic reactogenicity, or other unexpected adverse reactions that may arise due to changes to the vaccine strains composition following the annual WHO recommendations on the composition of influenza virus seasonal vaccines for the Northern hemisphere.^8^ This guidance outlines the requirements for influenza vaccine safety surveillance, with which all Marketing Authorization Holders (MAHs) providing vaccines in the EU must comply.^9^ The objective to enroll 1,000 vaccinated subjects covering different age-groups was made to comply with  the PRAC guidance for ESS.^8^ However, electronic health records (EHRs) offer the possibility to include higher numbers of subjects and thus to further stratify the findings by age or risk groups. Additionally, the data extracted, and coding of primarily pre-specified adverse events of interest (AEIs) are expected to be collected consistently, ultimately allowing for more robust year to year comparisons of results.

The present study reports the results of an ESS study of GSK’s inactivated quadrivalent influenza vaccine Fluarix Tetra, conducted in England during the 2017/18 northern-hemisphere influenza season.^10^ The study combined surveillance utilizing data from EHRs, enhanced by an adverse event reporting card (AERC), filled in by the vaccinees or their caregivers and returned to their General Practice (GP). The study was designed in compliance with the EMA requirements and with experience gained from two pilot studies conducted during the 2015/16 and 2016/17 seasonal influenza seasons.^11-13^

The objectives of the study were to estimate the weekly and cumulative incidence rates of AEIs following vaccination with a seasonal influenza vaccine, with a specific focus on Fluarix Tetra.

## Material and methods

This prospective ESS study (ClinicalTrials.gov number: NCT03278067) combined AERC (customized diary card) reported data and routinely collected medical data to provide the relevant information about influenza vaccine exposure (based on prescriptions) and safety. These data were collected and analyzed weekly and are presented in this manuscript as cumulative AEI incidence rate for the full study period (from 01-Sep-2017 to 30-Nov-2017). Adverse events/serious adverse events (SAEs) were reported to regulatory bodies according to local regulations throughout the study period.  A SAE (experience) or reaction is any untoward medical occurrence that at any dose: results in death, is life-threatening, requires inpatient hospitalisation or prolongation of existing hospitalisation, results in persistent or significant disability/incapacity, or is a congenital anomaly/birth defect.

### Setting and population

Ten GPs that used GSK’s Fluarix Tetra as their principal brand of influenza vaccine were enrolled, although other brands were also used. The decision to select the brand was the practices alone. Generally, practices order for the next season at the end of the previous season, so this decision would predate their involvement in the study. As the study was observational with no requests made to the GPs to recruit specific age or risk groups, the distribution of subject characteristics was dependent on GPs' routine practice. In order to maximise the possibility of enrolling subjects with a variety of characteristics, the GPs were geographically distributed across England.^11^ These practices are part of a Royal College of General Practitioners (RCGP) Research and Surveillance Centre (RSC) network, including 203 GP practices at the time of the study. Therefore, the frequency of AEs could be estimated using the same standardised coding system for events and analysis methodology for the EHR component. This also allowed for a similar data Quality Check and automated data extraction using standard tools such as Morbidity Information Query Export Syntax (MIQUEST).^14,15^

All individuals registered at the GPs who received a seasonal influenza vaccine (and had not opted out of data-sharing) were eligible for the study provided they had a valid and pseudonymised National Health Service (NHS) number, date of birth (simplified to year of birth on extract) and gender recorded in their EHR.

The study had no influence on vaccination practice, though it was anticipated that participating healthcare professionals follow the UK national flu immunization programme 2017/18 recommendations defined by JCVI.^6,16^ Since 2012, it is also recommended to vaccinate all children aged 2–8 years with an intranasal live attenuated influenza vaccine (LAIV), to both reduce transmission and provide direct protection. In 2017, the vaccination programme was extended to 9-year-old children and children 3–4 years old were offered the option to receive LAIV in reception class, rather than through their GP. Eligible adults aged 18 years and over had the choice of getting their inactivated influenza vaccine at a pharmacy within the Community Pharmacy Seasonal Influenza Vaccination Advanced Service.

One of the 10 GPs did not follow the protocol requirement to distribute AERC cards to all vaccinees. In addition, this GP destroyed the returned cards, and unused AERCs were not sent back to the University of Surrey which precludes any possibility to reliably estimate the events. Data from this practice were therefore excluded from the analyses.

Two GPs systematically recorded the date of onset of AEI as the date of data entry in the AERC. After verification, it appeared that only nine subjects out of 382 (2.4%) had AEIs that occurred after the 7-day post vaccination period in GSK’s Fluarix Tetra vaccine group. Therefore, taking a conservative approach, all reported events from these two practices were analyzed as occurring within the first 7 days post-vaccination.

### Data collection and extraction  from practice EHR systems

The following patient data were extracted for the study: (i) demographic information: age, gender, ethnicity, date of registration; (ii) Index of Multiple Deprivation (IMD) derived from the subjects’ postcodes;^17,18^ (iii) seasonal influenza vaccine information: date of administration, brand (Fluarix Tetra, other specified, unknown) and batch number as available; (iv) primary care consultations following vaccination  (7 days post vaccination), any other markers of health care utilization, and referral to further care; pre-specified AEIs or any other reported AE recorded in the EHR to allow comprehensive capture of events experienced by subjects; (v) data from at least one year prior to the start of the study to determine the category of UK Chief Medical Officer (CMO) specified risk group for influenza vaccination, and pregnancy status during the study period.

The method and governance procedure associated with the EHR system has been developed by the University of Surrey for the RCGP and RSC to meet the requirements for Public Health England for primary care surveillance.^14,15^

### Adverse events measures

#### Adverse events recorded via the adverse event reporting cards (enhanced component)

Subjects who received a seasonal influenza vaccine between 01-Sep-2017 to 30-Nov-2017 at their respective GP practice were provided with customized adverse event reporting cards (i.e., AERCs) to report AEIs within 7 days post vaccination. The cards included a list of pre-specified categories matching those AEIs specified by the EMA (Supplement 1).^8^ There was the option to specify if no AEI occurred or if other adverse reactions occurred within the specified timeframe. Study participants were asked to return the cards to the practice no later than 14 days following vaccination, by post or in person. The AEIs derived from the returned AERCs were entered in the EHR by practice staff.

#### Adverse events recorded via GP visits and pharmacy (EHR component)

AEs in vaccinees were extracted from the GP computer records of medically-attended visits, using a pre-defined code list (Supplement 2). The individual AEIs were categorized by Medical Dictionary for Regulatory Activities (MedDRA) system  organ class or relevant broad categories as follows: respiratory, gastrointestinal, fever, sensitivity or anaphylaxis, rash, other general symptoms, neurological, musculoskeletal and local symptoms related to vaccination.

Individuals who received their vaccination outside the practice (e.g., at a pharmacy) did not receive an AERC, however their records were extracted from EHR and they were included in the analysis.

### Statistical analysis

The number and proportion of individuals who received a seasonal influenza vaccine from 01-Sep-2017 to 30-Nov-2017 was calculated. Vaccinees were categorized as: vaccinated with Fluarix Tetra; vaccinated with a non-GSK brand of seasonal influenza vaccine; vaccinated with an unknown brand. Vaccinees were also categorized according to their age at vaccination, following the EMA PRAC guidance age groupings: 6 months to 5 years, 6 − 12 years, 13 − 17 years, 18 − 65 years, >65 years. Vaccinees were also categorized by UK CMO risk group status: asthma, chronic respiratory disease, chronic heart disease, chronic kidney disease, diabetes, immunosuppression (including relevant cancer treatment), chronic neurological disease, asplenia, pregnancy, 65 years old and older, or were categorized as not at risk. AEI categories are described in Supplement 2.

The primary objective of the ESS was to assess the weekly and cumulative incidence rate of any AEI reported by AERC within 7 days following seasonal influenza vaccination given between 01-Sep-2017 to 30-Nov-2017, with data by vaccine, age strata and UK CMO risk group status. Secondary objectives were to assess the weekly and cumulative incidence rate of any AEI reported by AERC as well as EHR record data from medically-attended visits within 7 days following seasonal influenza vaccination given between 01-Sep-2017 to 30-Nov-2017, with data by vaccine, age strata and UK CMO risk status. The outcomes for which data are not reported here can be found at Clinicaltrials.gov and GSK’s Study register.^19,20^ Cumulative AEI incidence stratified by age category and risk group status are presented from combined AERC and EHR data, for the most comprehensive approach.

The primary focus of this paper is on Fluarix Tetra, as the study was designed to assess the safety profile of this vaccine. While it is difficult to combine or compare data across different types of vaccines (such as LAIV and other IIV), the findings for non-GSK vaccines and for unknown vaccine brands are provided in the Supplemental files, along with weekly AEI incidence rates for Fluarix Tetra.

The 95% confidence intervals (CI) adjusting for clustering effect of GPs were computed on all estimated incidence rates (Clopper-Pearson exact CI modified for cluster data).^21^

R and statistical analysis system (SAS) software were used for the statistical analyses.

The cumulative incidence rates over the whole study period of specific AEIs corresponding with data in the Fluarix Tetra Summary of Product Characteristics (SmPC), were categorized as very common (≥1/10 or ≥10%), common (≥1/100 to <1/10 or ≥1% to <10%) and uncommon (≥1/1000 to <1/100 or ≥0.1% to <1%).^10^

### Ethical approval

The University of Surrey team sought the formal opinion of the Proportionate Review System of the National Ethics Review Service regarding the need for NHS Research Ethics Committee (REC) approval [REF: 17/NE/0286]. A waiver was granted consistent with the NHS National Research Ethics Service guidance.^22^

## Results

### Demographic data

The 9 eligible GPs were located in urban areas, each of them registering between approximately 8,000–16,000 patients. They were spread across England: North (2), Midlands and East (3), South (3) and London (1). The mean IMDs for subjects calculated by GP practice ranged from 8.3 to 26.1, with 0 representing the least deprived and 100 the most deprived score.

### Exposure data

**Figure 1.**
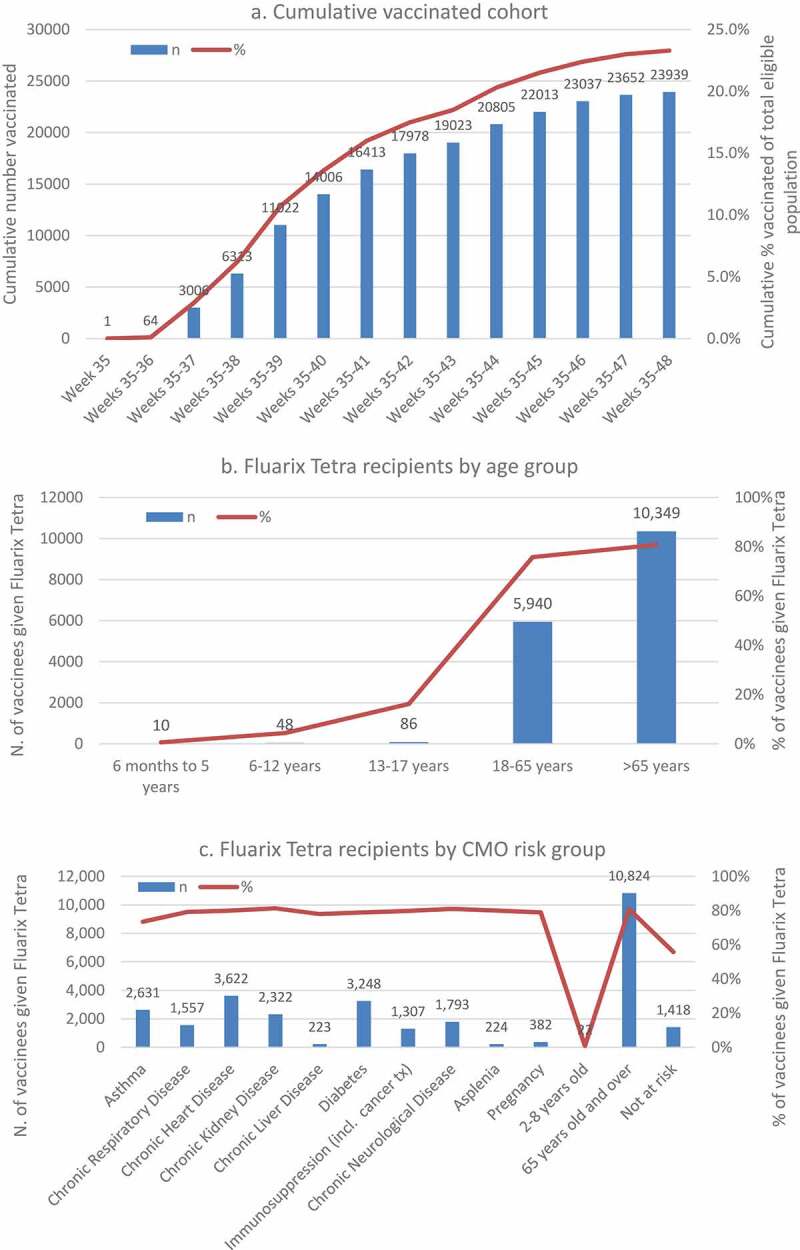
(a) cumulative vaccinated cohort (any vaccine) of total eligible population, and number (%) of fluarix tetra recipients by (b) age and (c) UK CMO-specified risk group.

There were 102,662 subjects registered with the nine eligible GPs, who did not opt out from data collection. Of these, 47 subjects were excluded because of an invalid NHS number, leaving 102,615 eligible subjects. Over the study period (01-Sep-2017 to 30-Nov-2017), 23,939 subjects were vaccinated (23.3%); 14 were not registered for post vaccination follow-up and therefore excluded. Figure 1 presents the cumulative number (%) of the eligible population vaccinated each week during the study. Most vaccinations took place between mid-September and mid-October (calendar weeks 37 to 41) (Figure 1).

CMO: Chief Medical Officer; incl.: including; n: number; tx: treatment; UK: United Kingdom.

Most vaccinated subjects received GSK’s Fluarix Tetra (68.6%, n = 16,433), 13.8% (n = 3,310) received other non-GSK vaccines and 17.5% (n = 4,196) received an unknown vaccine brand. The majority of Fluarix Tetra recipients were aged >65 years followed by 18–65 years. In the >65 years group and 18-65 years group, respectively,  10,349 subjects (80.8%) and 5,940 subjects (75.8%) received Fluarix Tetra. Fluarix Tetra was infrequently used in the pediatric population i.e., in 10 out of 1,661 pediatric vaccinees aged 6 months to 5 years, 48 out of 1,102 aged 6–12 years and 86 out of 532 aged 13–17 years. Regarding UK CMO risk groups, overall 70.2% of vaccinated at-risk subjects received GSK’s Fluarix Tetra compared with 55.8% of vaccinees not at risk. Figure 1 shows that around 80% of vaccinated subjects who were pregnant, diabetic, with asplenia or with a chronic condition (e.g., chronic respiratory, heart, kidney, liver or neurological condition) received Fluarix Tetra. Following local recommendations, the at-risk group aged 2–8 years primarily received other vaccines.

### Safety data

Of the 23,939 vaccinated subjects, 83.0% (n = 19,879) were given AERCs: 99.7%, 89.0% and 13.0% of Fluarix Tetra, non-GSK vaccine and unknown vaccine brand recipients, respectively. The return rate of AERCs was 30.8% (n = 5,053), 7.6% (n = 225) and 8.8% (n = 48) in the Fluarix Tetra, non-GSK vaccine and unknown vaccine brand groups, respectively.

Table 1 presents the AEIs reported, in Fluarix Tetra recipients, using AERCs alone and combining AERC data with EHR data from medically-attended visits or subjects vaccinated in Pharmacies. The cumulative incidence rate (% [95%CI]) of any AEIs reported within 7 days post-vaccination over the whole study was 7.25% [5.95–8.73] using AERCs and 9.21% [7.37–11.34] using AERC/EHR data (Table 1). The method of using AERC/EHR combined data identified some additional AEIs being reported (e.g., more muscle aches, cough, headache, rash), however, overall rates were comparable (overlapping 95% CIs) with AERC alone or AERC/EHR combined data. The most frequently reported categories of AEIs were respiratory symptoms 3.89% [3.12–4.79] and 4.70% [3.81–5.73], general nonspecific symptoms 2.56% (1.89; 3.38) and 2.82% [2.13–3.66] and, musculoskeletal symptoms 2.44% [1.96–3.00] and 3.05% [2.43–3.77], using AERCs and combined AERC/EHR data, respectively. The most frequently reported AEIs (cumulative incidence by combining AERC/EHR) were muscle aches (2.76% [2.20–3.42]), rhinorrhea (2.23% [1.58–3.05]), cough (1.94% [1.54–2.39]), headache (1.65% [1.14–2.31]), local symptoms (1.61% [1.20–2.11]), fatigue (1.52% [1.14–1.97]), oropharyngeal pain (1.33% [0.96–1.80]) and coryza (1.05% [0.80–1.35]). All other AEIs were reported with a cumulative incidence of <1% (Table 1). Cumulative incidence rates of AEIs for non-GSK and unknown brand vaccines are presented in Supplement 3 and weekly incidence rates are presented by vaccine in Supplement 4.

**Table 1.** Cumulative incidence rates of AEIs within 7 days post-vaccination reported by AERC or by AERC and EHR data combined, whole study period (Weeks 35–48)^a.^

	AERC, N = 16,387		AERC+EHR, N = 16,433	
AEIs	n	% [95%CI, LL-UL]	n	% [95%CI, LL-UL]
**Any AEIs**	**1,188**	**7.25 [5.95–8.73]**	**1,514**	**9.21 [7.37–11.34]**
**Local symptoms (i.e. local erythema)**	**259**	**1.58 [1.16–2.10]**	**264**	**1.61 [1.20–2.11]**
**Any general nonspecific symptoms**	**419**	**2.56 [1.89–3.38]**	**463**	**2.82 [2.13–3.66]**
Headache	244	1.49 [1.03–2.08]	271	1.65 [1.14–2.31]
Fatigue	228	1.39 [1.02–1.85]	249	1.52 [1.14–1.97]
<1%: Drowsiness	106	0.65 [0.32–1.16]	107	0.65 [0.33–1.16]
<1%: Fever/pyrexia	64	0.39 [0.23–0.62]	70	0.43 [0.25–0.67]
<1%: Irritability	38	0.23 [0.12–0.41]	41	0.25 [0.12–0.45]
<1%: Malaise	1	0.01 [0.00–0.04]	3	0.02 [0.00–0.05]
**Any sensitivity/anaphylaxis**	**62**	**0.38 [0.03–1.51]**	**68**	**0.41 [0.04–1.59]**
<1%: Rash	46	0.28 [0.20–0.39]	74	0.45 [0.32–0.62]
<1%: Anaphylactic reactions ^b^	42	0.26 [0.00–1.58]	45	0.27 [0.00–1.70]
<1%: Hypersensitivity reactions	16	0.10 [0.04–0.19]	18	0.11 [0.06–0.19]
<1%: Facial edema	7	0.04 [0.01–0.11]	8	0.05 [0.02–0.11]
**Any respiratory/miscellaneous**	**638**	**3.89 [3.12–4.79]**	**772**	**4.70 [3.81–5.73]**
Rhinorrhea	362	2.21 [1.57–3.01]	366	2.23 [1.58–3.05]
Cough	232	1.42 [1.06–1.85]	318	1.94 [1.54–2.39]
Oropharyngeal pain	203	1.24 [0.88–1.70]	219	1.33 [0.96–1.80]
Coryza	169	1.03 [0.80–1.30]	172	1.05 [0.80–1.35]
<1%: Nasal congestion	124	0.76 [0.53–1.04]	139	0.85 [0.60–1.15]
<1%: Hoarseness	99	0.60 [0.49–0.74]	104	0.63 [0.50–0.79]
<1%: Wheezing	70	0.43 [0.24–0.71]	81	0.49 [0.30–0.77]
<1%: Conjunctivitis	44	0.27 [0.18–0.39]	53	0.32 [0.20–0.49]
<1%: Epistaxis	10	0.06 [0.02–0.13]	14	0.09 [0.04–0.17]
**Any musculoskeletal**	**400**	**2.44 [1.96–3.00]**	**501**	**3.05 [2.43–3.77]**
Muscle aches/myalgia	353	2.15 [1.76–2.61]	454	2.76 [2.20–3.42]
<1%: Arthropathy	138	0.84 [0.55–1.23]	143	0.87 [0.57–1.27]
**Any gastrointestinal**	**217**	**1.32 [0.99–1.73]**	**248**	**1.51 [1.18–1.90]**
<1%: Nausea	115	0.70 [0.42–1.10]	118	0.72 [0.42–1.14]
<1%: Diarrhea	94	0.57 [0.44–0.73]	112	0.68 [0.55–0.84]
<1%: Decreased appetite	67	0.41 [0.27–0.60]	73	0.44 [0.30–0.64]
<1%: Vomiting	21	0.13 [0.08–0.20]	30	0.18 [0.12–0.27]
**Any neurological**	**25**	**0.15 [0.08–0.26]**	**28**	**0.17 [0.09–0.29]**
<1%: Peripheral tremor	24	0.15 [0.08–0.25]	26	0.16 [0.08–0.28]
<1%: Seizure/Febrile convulsions	1	0.01 [0.00–0.04]	1	0.01 [0.00–0.04]
<1%: Bell’s palsy	0	0.00 [0.00–0.02]	1	0.01 [0.00–0.04]
<1%: Guillain-Barre Syndrome	0	0.00 [0.00–0.02]	0	0.00 [0.00–0.02]

^a^Excludes results from GP failing to follow the protocol with respect to AERCs. Includes AERCs from two GPs systematically reporting AEI onset dates as AERC data entry dates. ^b^The code used to capture anaphylaxis was not specific enough so included all self-reported mild allergic reactions. No severe reactions were reported.

N: number of vaccinated subjects; n: number of subjects reporting the symptom at least once; % = (n/N)*100 = incidence rate of AEI; AEI: adverse event of interest; AERC: adverse event reporting card; EHR: electronic health record; 95% CI = 95% confidence interval (Clopper-Pearson exact CI modified for cluster data); LL = lower limit, UL = upper limit.

In Table 2, the incidence of AEIs in Fluarix Tetra recipients (from AERC/EHR combined data) is presented by age strata and UK CMO risk group status (categorized at risk/not at risk). The cumulative incidence rate (% [95%CI]) over the whole study was fairly consistent at 9.36% [7.29–11.78] and 9.19% [7.42–11.22] in the 18–65 year and the >65 year age groups, respectively. In the limited number of pediatric subjects who received Fluarix Tetra, rates of AEIs ranged from 2.33% [0.02–15.01] to 10.00% [0.14–48.28], with 1/10, 4/48 and 2/86 subjects in the age groups 6 months-5 years, 6–12 years and 13–17 years, respectively, reporting any AEIs. Incidence of AEIs was comparable for the at-risk and not at-risk groups (Table 2).

**Table 2.** Cumulative incidence rates of any AEI within 7 days post-vaccination, reported by AERC and EHR data combined, by age strata and UK CMO risk group, whole study period (Weeks 35–48).

	Any AEI incidence rate with Fluarix Tetra			
		N	n	% [95% CI, LL-UL]
**Age**	Any age	16,433	1,514	9.21 [7.37–11.34]
	6 months to 5 years	10	1	10.00 [0.14–48.28]
	6–12 years	48	4	8.33 [2.04–21.11]
	13–17 years	86	2	2.33 [0.02–15.01]
	18–65 years	5,940	556	9.36 [7.29–11.78]
	>65 years	10,349	951	9.19 [7.42–11.22]
**UK CMO-specified risk groups**	At risk	15,015	1,366	9.10 [7.28–11.20]
	Not at risk	1,418	148	10.44 [8.07–13.22]

N: number of vaccinated subjects; n: number of subjects reporting any AEI via AERC and EHR; % = (n/N)*100 = incident rate of any AEI; AEI: adverse event of interest; AERC: adverse event reporting card; CMO: Chief Medical Officer; UK: United Kingdom; 95% CI: 95% confidence interval (Clopper-Pearson exact CI modified for cluster data) LL: lower limit; UL: upper limit.

**Figure 2.**
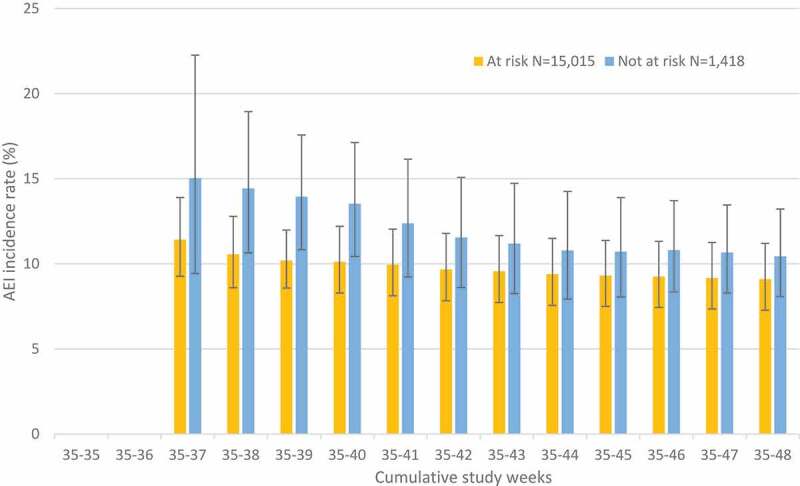
Cumulative AEI incidence rate (%) by study week, reported by AERC and EHR data combined for fluarix tetra, with 95% CI ^a^, stratified by subject UK CMO risk status.

^a^95% CI upper and lower limits provided when AEIs reported, from week 37 onwards.AEI: Adverse event of interest; AERC: Adverse event recording card; CMO: Chief Medical Officer; EHR: electronic health record; UK: United Kingdom; 95% CI: 95% Confidence interval (Clopper-Pearson exact CI modified for cluster data).

Figure 2 illustrates the week by week cumulative incidence rate of any AEI for Fluarix Tetra recipients (from AERC/EHR combined data), stratified by subjects’ UK CMO risk status categorized as at risk/not at risk. During the study period, as the number of subjects and events reported rises, precision around the incidence rate estimates increases. The overall cumulative incidence rate (% [95%CI]) for any AEI for at risk subjects was 9.10% [7.28–11.20] versus not at risk 10.44% [8.07–13.22], with overlap of the confidence intervals.

The AERC/EHR combined data for AEIs reported within 7 days of vaccination with Fluarix Tetra (all ages combined due to limited pediatric data) are presented with the reporting rates of the corresponding events in the SmPC (Table 3).^10^ SmPC safety data, derived primarily from clinical trial data with high levels of reporting and follow up, were used as a benchmark for expected reporting rates. The events of this study, when categorized as very common, common or uncommon, were reported in an equal or lower category compared with the SmPC safety data (e.g., while fever was identified as a common AEI in a clinical trial setting, it was identified as an uncommon AEI in the context of this study). An analysis by Fluarix Tetra batch was not conducted since no safety signal was detected.

**Table 3.** Frequency of AEIs from AERC and EHR data combined (all ages) and summary of product characteristics solicited events by age group.

	Observed frequency SmPC by age group				Observed frequency ESS
AEIs	6mo–3y	3–6y	6–18y	≥18y	All age groups
**General disorders and administration site conditions**					*% [95%CI, LL-UL]*
Fever	Common	Common	Common	Common	Uncommon *0.43 [0.25–0.67]*
Fatigue	N/A	N/A	Very common	Very common	Common *1.52 [1.14–1.97]*
Injection site redness	Very common	Very common	Very common	Common	Common^a^ *1.61 [1.20–2.11]*
**Nervous system disorders**					
Headache	N/A	N/A	Common	Common	Common *1.65 [1.14–2.31]*
Drowsiness	Very common	Common	N/A	Uncommon	Uncommon *0.65 [0.33–1.16]*
**Psychiatric disorders**					
Irritability	Very common	Very common	N/A	N/A	Uncommon *0.25 [0.12–0.45]*
**Musculoskeletal disorders**					
Myalgia	N/A	N/A	Very common	Very common	Common *2.76 [2.20–3.42]*
Arthralgia	N/A	N/A	Common	Common	Uncommon *0.87 [0.57–1.27]*
**Gastrointestinal disorders**					
Nausea	N/A	N/A	Common	Common	Uncommon *0.72 [0.42–1.14]*
Vomiting	N/A	N/A	Common	Common	Uncommon *0.18 [0.12–0.27]*
Diarrhea	N/A	N/A	Common	Common	Uncommon *0.68 [0.55–0.84]*
**Metabolism and nutrition disorders**					
Loss of appetite	Very common	Common	N/A	N/A	Uncommon *0.44 [0.30–0.64]*
**Skin and subcutaneous tissue disorders**					
Rash	N/R	Uncommon	Uncommon	N/A	Uncommon *0.45 [0.32–0.62]*

AEIs for Fluarix Tetra in the different age groups are listed per dose according to the following frequency categories in the SmPC:^10^ Very common: ≥1/10; Common: ≥1/100 to <1/10; Uncommon: ≥1/1,000 to <1/100; Rare: ≥1/10,000 to <1/1,000; Very rare: <1/10,000

^a^In the study this event was captured under local erythema; AEI: Adverse event of interest; AERC: Adverse event recording card; EHR: electronic health record; ESS: enhanced safety surveillance; N/A = Not solicited in this age group; N/R = Not reported; SmPC: Summary of Product Characteristics; 95% CI: 95% Confidence interval (Clopper-Pearson exact CI modified for cluster data); LL: lower limit; UL: upper limit.

Of note, a total of 38 subjects (0.16%) reported SAEs within the 7 days post-vaccination period, 25 were among Fluarix Tetra’s recipients, 3 in non-GSK vaccine group, and 10 in the unknown vaccine brand groups. None of the SAEs were considered related to vaccination. (Of note, no SAEs were reported within the 7 days post vaccination period for the GP practice excluded from safety analyses.) The single event SAEs reported for Fluarix Tetra were abdominal pain, asthma, blood transfusion, cancer, COPD, bone fracture, frailty, gynaecological, head injury, chronic wound infection, sensitivity reaction and stroke. There were three SAEs reported as chest pain and five SEAs reported as accidental fall and unknown/non-causal. The single event SAEs reported for the non-GSK vaccine group were atrial fibrillation, cancer and chest pain. The single event SAEs reported in the unknown vaccine brand groups were abdominal pain, accidental fall, chronic ear infection, hyperkalaemia, hypoglycaemia, chronic wound infection, pneumonia, self-harm, unknown/non-causal and viral gastroenteritis.

## Discussion

Following the removal of the requirement for pre-registration clinical trials of seasonal influenza vaccines, the EMA PRAC suggested three options for vaccine manufacturers to monitor AEIs following vaccination,^8^ namely: active surveillance using existing methods of post-authorization studies; ESS in which vaccine coverage is rapidly estimated and additional steps are taken to facilitate recording of AEIs; use of EHR with data mining. The objective is for MAHs to detect any potential safety concerns associated with the vaccine in near real-time following an update of seasonal vaccine strain composition.

In response, GSK, in conjunction with the University of Surrey, began to implement ESS in 2015 in England, where medical records are computerized enabling access to primary care and vaccination data. With a customized adverse reaction reporting card to enhance reporting rates of AEIs via EHRs, the present study estimated the cumulative incidence rate of any AEI within 7 days following seasonal influenza vaccination given between 01-Sep-2017 to 30-Nov-2017, by age strata and UK CMO risk group status.

To capture AEIs post-vaccination, EHR-based surveillance was combined with AERCs for an enhanced approach. As specified in EMA guidance (section 2.4.2),^8^ the principle of enhanced surveillance is to rapidly estimate vaccine usage (number vaccinated or doses administered) and to facilitate passive ADR reporting, in order to derive reporting rates as a surrogate of AEI incidence. The importance of using existing regional frameworks (e.g., influenza sentinel surveillance networks) to gather relevant data was also highlighted.

This type of study complements existing safety monitoring strategies, with the aim of early identification of safety signals and maximizing prompt investigation of any signals detected. This initiative helps to raise awareness, with patients and physicians, of the importance of actively reporting AEs after vaccination.

The cumulative incidence rates of AEIs for Fluarix Tetra were 7.25% [5.95–8.73] for events reported by card alone, and 9.21% [7.37–11.34] when combining EHR data. The most frequently reported events were respiratory symptoms, musculoskeletal symptoms and general nonspecific symptoms. The rates of occurrence were of similar magnitude or lower than reported in the Fluarix Tetra SmPC (Table 3) and higher than reported by the RCGP RSC network of EHR data from 203 GP practices, where the AEI rate for Fluarix Tetra (26,249 vaccinees) was 5.40% [3.73–7.52].^10^ The reporting rate differences were anticipated considering the methods of reporting: highest for the SmPC rates that are derived from a solicited approach in clinical trials (e.g., active surveillance, reminders or active follow-up of non-responders, longer follow-up duration) and lowest for the RCGP RSC network which relies essentially on spontaneous reporting, as compared to the current study in which an enhanced approach was used.

Approximately three quarters of the AEIs were reported via returned AERCs, suggesting the enhancement was a feasible and effective approach to address the EMA requirements (assuming the cards were completed accurately and within the prescribed time-period). However, the rates of card return varied across the vaccination groups: 30.84% of cards were returned for Fluarix Tetra, 7.64% for non-GSK brands and 8.79% for unknown brands.

Vaccination uptake differed according to age groups as well as the distribution of brand used, which is consistent with local recommendations, in which children were to be offered LAIV preferentially while in adults inactivated vaccines were to be used. Fluarix Tetra was primarily given to older adults (10,349 of 16,433 vaccinees (62.97%) were 65 years old and over). There are no clear explanations for the difference in response rates between adults/older adults (receiving IIV) and children (receiving LAIV). The participating sites were aware that the focus of the study was on Fluarix Tetra, therefore it cannot be ruled out that they used a slightly different approach depending on the vaccine administered, although this is unlikely given the instructions provided. As few children were given Fluarix Tetra, it is difficult to compare AERC response rates by age group for IIV recipients. Even if GPs were instructed to apply the same approach to all enrolled subjects regardless of the vaccine brand received, AERCs were not commonly used in the pediatric population, likely because children were offered vaccination at school rather than through general practice.^6^ As compared to children, reporting rates among elderly subjects are consistent with previous findings, suggesting that the number of adverse reactions to medication may increase with age (with frailty, medical history and concomitant use of medications),^23^ and a similar trend was observed in an ESS of influenza vaccines published previously.^24^ LAIV is administered intra-nasally while IIV by intramuscular injection, this could result in a different reactogenicity profile, which may result in fewer AEIs and thus a lower probability of reporting an AEI, seeking a medical visit or returning the AERC.

Although influenza vaccination rates in England are relatively high compared with other European countries,^25^ the pediatric age-group was underrepresented in this study as a consequence of the JVCI preferential recommendation to use intranasal LAIV in children aged 2–8 years in England and of school vaccination programs rather than in GP settings.^6^ In the future, this limitation could be mitigated by including a country in which no preferential recommendations are applied for the pediatric population or by specifically targeting pediatric centers, and thus where Fluarix Tetra is used broadly in children, including healthy individuals. This approach has recently been adapted and is expected to show promising results.^26^

In this study the subjects enrolled were registered to urban GP practices. Since the IMDs of subjects were not representative of the country as a whole, the generalizability of the results is potentially limited. The study did not capture information relating to co-administration of vaccines. It cannot be excluded that additional vaccines were co-administered (for example zoster or pneumococcal vaccines) so events attributed to influenza vaccination specifically may have been over-reported. In addition, it is acknowledged that a seven-day timeframe is not sufficiently long to adequately capture some events, such as Guillain-Barre syndrome or Bell’s palsy, which can occur after several weeks. This time period was however, specified by the EMA for these studies, and is expected to be comparable with other data collected. Another limitation is that there is no control over actual reporting rates, under-reporting of events could still be a possibility. The customized AERC used in this study contained the option to record ‘no adverse events’ potentially improving return rates, however the use of a dedicated free-phone telephone line with systematic interview, as in a recent ESS,^24^ and/or a web-based alternative could possibly improve the response rates for the young adult age-group.

A strength of this study was its size; ~24,000 vaccinees were included with approximately two-thirds vaccinated with Fluarix Tetra. The size of the study was sufficient to capture very common (≥1/10), common (≥1/100 to <1/10) and uncommon (≥1/1000 to <1/100) events. Stratified analysis was also possible to explore safety endpoints by age and by risk status with the population enrolled essentially covering the population targeted in England’s vaccination recommendations. The AERC return rate was relatively limited. In future, additional strategies can be envisaged to maximize subject response rates, including but not limited to surveys, using an application over a web-based system, automated reminders during follow-up or a scheduled visit.^27^ The reporting rates, on the other hand, were very similar between risk groups and non-risk groups which confirm the safety profile of Fluarix Tetra regardless of the health status of vaccinees. The standardized approach to reporting and data extraction is expected to provide stability and consistency to enable future comparisons.

In conclusion, this study highlights the added value of combining the EHR record system with customized cards to better capture adverse events occurring post vaccination. This study did not identify any safety signal which could impact public health or alter the benefit-risk profile. The study supports and confirms the safety profile of Fluarix Tetra.

## Supplementary Material

Supplemental Material

## Trademark statement

Fluarix Tetra is a trademark of the GSK group of companies.
