# Supplementary material for: Brand-specific enhanced safety surveillance of GSK’s Fluarix Tetra seasonal influenza vaccine in England: 2017/2018 season
Source: Hum Vaccin Immunother. 2020 Mar 2;16(8):1762–71. doi: 10.1080/21645515.2019.1705112 (PMC7482908; doi:10.1080/21645515.2019.1705112)
Supplement: Supplemental Material [file KHVI_A_1705112_SM3541.zip › Supplement 1.pdf]

## Supplement 1. Adverse event reporting card

gsk GlaxoSmithKline — Version 1 – May 15th, 2017 — UNIVERSITY OF SURREY

**CONFIDENTIAL** Card unique number: 0000 0000 0000 0

### Enhanced safety surveillance of seasonal influenza (flu) vaccine

Study of possible adverse events following immunisation – this surveillance is designed to capture all adverse events following immunisation. Please report if you get any symptoms following your "flu jab" (influenza vaccination).

**1. About you – this information is kept confidential and won't leave your practice**

**About you*** we need contact details, please supply a full address so we can link this to your medical record:

First name ___________________ Surname ________________________________

Address ___________________________________________________________________________

Postcode __________ Telephone: ____________ Email _________@________________

Signed _______________________ Date ___ /____ /2017

***This personal information is only being collected to link any side effects to your record**

**2. When you were vaccinated / When was the influenza vaccination given**

**What date were you vaccinated / was the vaccine given** ___ /____ /2017

**Where were you vaccinated:** At your GP surgery: ☐ Yes ☐ No If no, say where: ___________

**3. If you were not the person vaccinated**

**Information about the person*** ☐ Male ☐ Female Date of Birth ___ /____ /___

First name ___________________ Surname ________________________________

**4. Please report any side-effects/conditions in the 7 days after your flu vaccine**

**Please look at the list of possible vaccine side-effects on the next page – if** *the person vaccinated* **has experienced any adverse events – please tick the relevant box and indicate the severity**

Please also mark if the symptoms/possible side effects are still persisting

*Please return the card in the envelope provided to your GP – please return by post or in person.*

*Thank you for your help*

**5. If you had no side effects in the 7 days after vaccination tick and return**

**I /the person vaccinated has NOT had any side effects or other symptoms following vaccination:** ☐

*Please return the card in the envelope provided to your GP – please return by post or in person.*

*Thank you for your help*

## Enhanced safety surveillance of seasonal influenza vaccine

| Possible side effect or Condition in the 7 days after influenza vaccination | Start date of the symptom | Please tick as appropriate |
|---|---|---|
| Conjunctivitis – Sticky eyes | ___/___/17 | ☐ |
| Runny nose | ___/___/17 | ☐ |
| Blocked nose | ___/___/17 | ☐ |
| Epistaxis – Nose bleed | ___/___/17 | ☐ |
| Common cold | ___/___/17 | ☐ |
| Cough | ___/___/17 | ☐ |
| Sore throat | ___/___/17 | ☐ |
| Hoarse voice | ___/___/17 | ☐ |
| Wheezing | ___/___/17 | ☐ |
| Decreased appetite | ___/___/17 | ☐ |
| Nausea – feeling sick | ___/___/17 | ☐ |
| Vomiting – being sick | ___/___/17 | ☐ |
| Diarrhoea | ___/___/17 | ☐ |
| Fever (add temperature if measured) | ___/___/17 | ☐ |
| Allergic reaction (rash) | ___/___/17 | ☐ |
| Other allergic reactions | ___/___/17 | ☐ |
| Facial oedema (swelling) | ___/___/17 | ☐ |
| Local reaction to vaccine | ___/___/17 | ☐ |
| Rash | ___/___/17 | ☐ |
| Irritability | ___/___/17 | ☐ |
| Drowsiness | ___/___/17 | ☐ |
| Fatigue | ___/___/17 | ☐ |
| Tremor / shaking | ___/___/17 | ☐ |
| Seizure / fits | ___/___/17 | ☐ |
| Headache | ___/___/17 | ☐ |
| Muscle aches | ___/___/17 | ☐ |
| Joint pain | ___/___/17 | ☐ |
| Other | | |
| 1. ____________________ | ___/___/17 | ☐ |
| 2. ____________________ | ___/___/17 | ☐ |
| 3. ____________________ | ___/___/17 | ☐ |
| Add below if more | | |
