# Supplementary material for: Brand-specific enhanced safety surveillance of GSK’s Fluarix Tetra seasonal influenza vaccine in England: 2017/2018 season
Source: Hum Vaccin Immunother. 2020 Mar 2;16(8):1762–71. doi: 10.1080/21645515.2019.1705112 (PMC7482908; doi:10.1080/21645515.2019.1705112)
Supplement: Supplemental Material [file KHVI_A_1705112_SM3541.zip › Supplement 2.pdf]

# Supplement 2. Code list of pre-specified AEIs

| Pre-specified surveillance condition | Read Code (5 Byte) | Read Code (CTV3) | Notes |
|---|---|---|---|
| **Respiratory/Miscellaneous** | | | |
| Conjunctivitis | F4C0. | XE16X | Sticky eyes |
| Rhinorrhoea | 1C83. | XM00h | Runny nose |
| Nasal congestion | H1y1z | X77Gp | Blocked nose |
| Epistaxis | R047. | Xa96W | Nose bleed |
| Coryza | H00.. | XE0Xl | Common cold |
| Cough | 171.. | XM0Ch | |
| Oropharyngeal pain | 1922.<br>1CB3. | 1922.<br>1CB3. | Sore throat |
| Hoarseness | 1CA2. | 1CA2. | Hoarse voice |
| Wheezing | 1737. | XE0qs | |
| **Gastrointestinal** | | | |
| Decreased appetite | R0300 | XM07Y | |
| Nausea | 198.. | X75qw | Feeling sick |
| Vomiting | 199.. | XE0rA | Being sick |
| Diarrhoea | 19F.. | 19F2. | |
| **Fever/pyrexia** | | | |
| Fever | 165.. | X76Dl | |
| Mild fever (<38.5° C rectal) | 2E3.. | 2E3.. | Please include level of temperature, to help us classify the fever |
| Moderate fever (38.6-39.5°C) | | | |
| High fever (>39.5°C) | | | |
| **Sensitivity/anaphylaxis** | | | |
| Hypersensitivity reactions | SN52. | Xa5uf | Allergic reaction |
| Anaphylactic reactions | SN501 | X70vr | Other allergic reactions |
| Facial oedema | 16J5. | Xa0ls | Facial swelling |
| **Rash** | | | |
| Rash | M130. | X50Ge | |
| Generalised rash | 2I14. | XM07J | |
| **General non-specific symptoms** | | | |
| Irritability | 225A. | 225A. | |
| Drowsiness | 1B67. | XM06R | |
| Fatigue | 168.. | 1682. | |
| Headache | 1B1G. | XM0CV | |
| **Neurological** | | | |
| Bell's palsy | F310. | F310. | |
| Peripheral tremor | 1B22. | XE0rn | Tremor/shaking |
| Guillain-Barre Syndrome (GBS) | F3700 | F3700 | |
| Seizure/ Febrile convulsions | 1B64.<br>1B6B. | XaDbE<br>XM03l | Seizure/ fits |
| **Musculoskeletal** | | | |
| Muscle aches/ myalgia | N2410 | X75rs | |
| Arthropathy | N037. | X701f | Joint pain |
| **Local Symptoms** | | | |
| Local erythema | SP3y4<br>SP3y5<br>SP3y6<br>SP3y7 | X75ty | Local reaction to vaccine |
