# Supplementary material for: Brand-specific enhanced safety surveillance of GSK’s Fluarix Tetra seasonal influenza vaccine in England: 2017/2018 season
Source: Hum Vaccin Immunother. 2020 Mar 2;16(8):1762–71. doi: 10.1080/21645515.2019.1705112 (PMC7482908; doi:10.1080/21645515.2019.1705112)
Supplement: Supplemental Material [file KHVI_A_1705112_SM3541.zip › Supplement 3.docx]

## Supplement 3

## Cumulative incidence rates of AEIs within the 7 days post-vaccination period reported via AERC, by vaccine group, over the whole study period (Weeks 35-48) ^a^

| **AEIs** | **Non-GSK vaccines, N = 2,946** | | **Unknown vaccine brands, N = 546** | |
| --- | --- | --- | --- | --- |
|  | **n** | **% [95% CI, LL-UL]** | **n** | **% [95% CI, LL-UL]** |
| **Any AEIs** | **75** | **2.55 [1.01-5.23]** | **11** | **2.01 [1.01-3.58]** |
| **Local symptoms (i.e. local erythema)** | **17** | **0.58 [0.12-1.68]** | **1** | **0.18 [0.00-1.57]** |
| **Any general non-specific symptoms** | **26** | **0.88 [0.20-2.48]** | **3** | **0.55 [0.11-1.60]** |
| Headache | 17 | 0.58 [0.13-1.64] | 2 | 0.37 [0.04-1.32] |
| Fatigue | 10 | 0.34 [0.10-0.84] | 2 | 0.37 [0.04-1.32] |
| Fever/pyrexia | 8 | 0.27 [0.05-0.80] | 1 | 0.18 [0.00-1.02] |
| Drowsiness | 7 | 0.24 [0.03-0.83] | 0 | 0.00 [0.00-0.67] |
| Irritability | 4 | 0.14 [0.01-0.62] | 0 | 0.00 [0.00-0.67] |
| Malaise | 0 | 0.00 [0.00-0.13] | 0 | 0.00 [0.00-0.67] |
| **Any sensitivity/ anaphylaxis** | **7** | **0.24 [0.01-1.08]** | **1** | **0.18 [0.00-1.02]** |
| Anaphylactic reactions ^b^ | 7 | 0.24 [0.01-1.08] | 1 | 0.18 [0.00-1.02] |
| Rash | 1 | 0.03 [0.00-0.19] | 0 | 0.00 [0.00-0.67] |
| Facial oedema | 0 | 0.00 [0.00-0.13] | 0 | 0.00 [0.00-0.67] |
| Hypersensitivity reactions | 0 | 0.00 [0.00-0.13] | 0 | 0.00 [0.00-0.67] |
| **Any respiratory/miscellaneous** | **44** | **1.49 [0.73-2.69]** | **6** | **1.10 [0.40-2.38]** |
| Rhinorrhoea | 28 | 0.95 [0.47-1.70] | 3 | 0.55 [0.11-1.60] |
| Cough | 22 | 0.75 [0.31-1.52] | 1 | 0.18 [0.00-1.02] |
| Coryza | 11 | 0.37 [0.13-0.85] | 1 | 0.18 [0.00-1.73] |
| Oropharyngeal pain | 9 | 0.31 [0.06-0.91] | 2 | 0.37 [0.04-1.32] |
| Wheezing | 9 | 0.31 [0.08-0.80] | 0 | 0.00 [0.00-0.67] |
| Nasal congestion | 8 | 0.27 [0.11-0.56] | 2 | 0.37 [0.04-1.32] |
| Hoarseness | 5 | 0.17 [0.03-0.55] | 1 | 0.18 [0.00-1.02] |
| Conjunctivitis | 3 | 0.10 [0.02-0.30] | 2 | 0.37 [0.04-1.32] |
| Epistaxis | 0 | 0.00 [0.00-0.13] | 0 | 0.00 [0.00-0.67] |
| **Any musculoskeletal** | **12** | **0.41 [0.02-1.84]** | **4** | **0.73 [0.13-2.24]** |
| Muscle aches / myalgia | 12 | 0.41 [0.02-1.84] | 3 | 0.55 [0.11-1.60] |
| Arthropathy | 6 | 0.20 [0.01-0.92] | 1 | 0.18 [0.00-1.71] |
| **Any gastrointestinal** | **13** | **0.44 [0.15-1.00]** | **2** | **0.37 [0.04-1.32]** |
| Decreased appetite | 6 | 0.20 [0.07-0.45] | 0 | 0.00 [0.00-0.67] |
| Nausea | 4 | 0.14 [0.04-0.35] | 1 | 0.18 [0.00-1.02] |
| Diarrhoea | 2 | 0.07 [0.00-0.31] | 1 | 0.18 [0.00-1.02] |
| Vomiting | 1 | 0.03 [0.00-0.19] | 1 | 0.18 [0.00-1.02] |
| **Any neurological** | **2** | **0.07 [0.00-0.31]** | **1** | **0.18 [0.00-1.02]** |
| Peripheral tremor | 2 | 0.07 [0.00-0.31] | 0 | 0.00 [0.00-0.67] |
| Seizure / Febrile convulsions | 0 | 0.00 [0.00-0.13] | 1 | 0.18 [0.00-1.02] |
| Bell’s palsy | 0 | 0.00 [0.00-0.13] | 0 | 0.00 [0.00-0.67] |
| Guillain-Barre Syndrome | 0 | 0.00 [0.00-0.13] | 0 | 0.00 [0.00-0.67] |

^a^ Excludes results from GP failing to follow the protocol with respect to AERCs. Includes all AERCs for the two GPs systematically reporting AEIs onset dates as the AERC data entry dates. ^b^ The code used to capture anaphylaxis was not specific enough (refer to Supplementary table 2) so included all self-reported mild allergic reactions. No severe reactions were reported.

N = number of vaccinated subjects; n = number of subjects reporting the symptom at least once; % = (n/N)*100 = incidence rate of AEI; AEI: adverse event of interest; AERC: adverse event reporting card; 95% CI = 95% confidence interval (Clopper-Pearson exact CI modified for cluster data); LL = lower limit, UL = upper limit
