# Supplementary material for: Brand-specific enhanced safety surveillance of GSK’s Fluarix Tetra seasonal influenza vaccine in England: 2017/2018 season
Source: Hum Vaccin Immunother. 2020 Mar 2;16(8):1762–71. doi: 10.1080/21645515.2019.1705112 (PMC7482908; doi:10.1080/21645515.2019.1705112)
Supplement: Supplemental Material [file KHVI_A_1705112_SM3541.zip › Supplement 4.docx]

**Supplement 4**

**Weekly incidence rates of any AEIs within 7 days post-vaccination reported by AERC, by vaccine group, whole study period (weeks 35-48)**

| **Week** | **GSK’s Fluarix Tetra** | | | **Non-GSK vaccines** | | | **Unknown vaccine brands** | | | **All vaccinated** | | |
| --- | --- | --- | --- | --- | --- | --- | --- | --- | --- | --- | --- | --- |
|  | **N** | **n** | **% [95% CI, LL-UL]** | **N** | **n** | **% [95% CI, LL-UL]** | **N** | **n** | **% [95% CI, LL-UL]** | **N** | **n** | **% [95% CI, LL-UL]** |
| 35 | 0 | - | - | 0 | - | - | 0 | - | - | 0 | - | - |
| 36 | 1 | 0 | 0.00 [0.00-97.50] | 1 | 0 | 0.00 [0.00-97.50] | 3 | 0 | 0.00 [0.00-70.76] | 5 | 0 | 0.00 [0.00-52.18] |
| 37 | 2,466 | 249 | 10.10 [8.53-11.84] | 3 | 0 | 0.00 [0.00-70.76] | 16 | 2 | 12.50 [1.55-38.35] | 2,485 | 251 | 10.10 [8.58-11.79] |
| 38 | 2,730 | 210 | 7.69 [5.94-9.77] | 7 | 1 | 14.29 [0.00-90.97] | 16 | 2 | 12.50 [1.50-38.73] | 2,753 | 213 | 7.74 [5.95-9.86] |
| 39 | 4,116 | 328 | 7.97 [7.16-8.84] | 94 | 0 | 0.00 [0.00-3.85] | 24 | 1 | 4.17 [0.11-21.12] | 4,234 | 329 | 7.77 [6.98-8.62] |
| 40 | 2,055 | 169 | 8.22 [4.92-12.73] | 337 | 16 | 4.75 [2.74-7.60] | 31 | 0 | 0.00 [0.00-11.22] | 2,423 | 185 | 7.64 [4.69-11.61] |
| 41 | 1,657 | 106 | 6.40 [4.45-8.86] | 338 | 11 | 3.25 [1.08-7.37] | 56 | 1 | 1.79 [0.05-9.55] | 2,051 | 118 | 5.75 [3.99-8.00] |
| 42 | 910 | 34 | 3.74 [1.74-6.93] | 401 | 5 | 1.25 [0.36-3.06] | 17 | 0 | 0.00 [0.00-19.51] | 1,328 | 39 | 2.94 [1.59-4.94] |
| 43 | 496 | 23 | 4.64 [1.86-9.38] | 378 | 10 | 2.65 [0.69-6.81] | 22 | 0 | 0.00 [0.00-15.44] | 896 | 33 | 3.68 [1.92-6.34] |
| 44 | 750 | 33 | 4.40 [1.33-10.39] | 464 | 17 | 3.66 [0.70-10.72] | 30 | 0 | 0.00 [0.00-11.57] | 1,244 | 50 | 4.02 [1.47-8.61] |
| 45 | 453 | 17 | 3.75 [1.64-7.25] | 402 | 9 | 2.24 [0.20-8.71] | 261 | 5 | 1.92 [0.62-4.41] | 1,116 | 31 | 2.78 [1.38-4.96] |
| 46 | 350 | 15 | 4.29 [1.59-9.10] | 264 | 5 | 1.89 [0.08-9.13] | 19 | 0 | 0.00 [0.00-17.65] | 633 | 20 | 3.16 [1.08-7.05] |
| 47 | 258 | 4 | 1.55 [0.22-5.19] | 157 | 1 | 0.64 [0.02-3.50] | 41 | 0 | 0.00 [0.00-8.60] | 456 | 5 | 1.10 [0.23-3.15] |
| 48 | 145 | 0 | 0.00 [0.00-2.51] | 100 | 0 | 0.00 [0.00-3.62] | 10 | 0 | 0.00 [0.00-30.85] | 255 | 0 | 0.00 [0.00-1.44] |

N = number of subjects; n = number of subjects reporting the symptom at least once on the AERC; % = (n / N) X 100

For GPs 5 and 10, it was assumed that all reported AEIs with onset date as the AERC data entry date occurred within the 7 days post-vaccination period

AERC: Adverse event recording card; AEI: Adverse event of interest; 95% CI: 95% Confidence interval (Clopper-Pearson exact CI modified for cluster data); LL: lower

limit, UL: upper limit
